# Entomological surveillance of behavioural resilience and resistance in residual malaria vector populations

**DOI:** 10.1186/1475-2875-12-124

**Published:** 2013-04-11

**Authors:** Nicodem J Govella, Prosper P Chaki, Gerry F Killeen

**Affiliations:** 1 Ifakara Health Institute, Environmental Health and Ecological Sciences Thematic Group, P.O. Box 78373, Dar es Salaam, United Republic of Tanzania; 2 Vector Biology Department, Liverpool School of Tropical Medicine, Pembroke Place, Liverpool, L3 5QA, UK

**Keywords:** Malaria, Transmission, Interventions, Mosquito, Behaviour, Resistance, Resilience, Phenotypic plasticity, Genetic

## Abstract

**Background:**

The most potent malaria vectors rely heavily upon human blood so they are vulnerable to attack with insecticide-treated nets (ITNs) and indoor residual spraying (IRS) within houses. Mosquito taxa that can avoid feeding or resting indoors, or by obtaining blood from animals, mediate a growing proportion of the dwindling transmission that persists as ITNs and IRS are scaled up.

**Presentation of the hypothesis:**

Increasing frequency of behavioural evasion traits within persisting residual vector systems usually reflect the successful suppression of the most potent and vulnerable vector taxa by IRS or ITNs, rather than their failure. Many of the commonly observed changes in mosquito behavioural patterns following intervention scale-up may well be explained by modified taxonomic composition and expression of phenotypically plastic behavioural preferences, rather than altered innate preferences of individuals or populations.

**Testing the hypothesis:**

Detailed review of the contemporary evidence base does not yet provide any clear-cut example of true behavioural resistance and is, therefore, consistent with the hypothesis presented.

**Implications of the hypothesis:**

Caution should be exercised before over-interpreting most existing reports of increased frequency of behavioural traits which enable mosquitoes to evade fatal contact with insecticides: this may simply be the result of suppressing the most behaviourally vulnerable of the vector taxa that constituted the original transmission system. Mosquito taxa which have always exhibited such evasive traits may be more accurately described as behaviourally *resilient*, rather than *resistant*. Ongoing national or regional entomological monitoring surveys of physiological susceptibility to insecticides should be supplemented with biologically and epidemiologically meaningfully estimates of malaria vector population dynamics and the behavioural phenotypes that determine intervention impact, in order to design, select, evaluate and optimize the implementation of vector control measures.

## Background

Existing front line tools for malaria vector control, namely insecticide-treated nets (ITNs) and indoor residual spraying (IRS), have greatly reduced the malaria burden [1,2] because the most important mosquito vectors feed predominantly upon people at times when they are inside their houses so that insecticidal contact is maximized [3-5]. These synanthropic vectors can be described as being behaviourally vulnerable to control with such indoor applications of insecticides because it is possible to achieve high coverage of the blood and resting site resources they need to survive. Both recent and historical reports from sub-Saharan Africa show that widespread use of ITNs or IRS change the species composition [6-13] of residual vector populations by progressively diminishing densities of each species in proportion to its physiological susceptibility to insecticides [14], their behavioural vulnerability to insecticide contact [15-17] arising from their propensity to feed (endophagic) or rest (endophilic) indoors [9,18-20], and their preference for human blood (anthropophagic) [7]. For example, the widespread and exceptionally efficient African vector *Anopheles funestus*, which feeds almost exclusively upon human blood and predominantly feeds and rests indoors [6,21], was eliminated from the Pare-Taveta study area in Tanzania during the 1960s following three years of IRS with dieldrin [10]. This species took six years to re-establish itself in the area, during which time it was replaced by *Anopheles rivulorum* and *Anopheles parensis,* two morphologically similar species from the same group that prefer to feed outdoors (exophagic) and are generally thought to be of secondary relevance to transmission because they prefer to obtain blood from animals (zoophagic) [6,10]. In South Africa, *An. funestus* was eliminated from the entire country by IRS with DDT in the 1950s [22] and was successfully excluded for half a century when a switch to pyrethroids allowed re-invasion by physiologically resistant populations [23]. In the Solomon Islands, IRS and ITN have eliminated *Anopheles koliensis*, while *Anopheles punctulatus* is now increasingly uncommon with a patchy distribution, leaving only *Anopheles farauti* as the sole primary vector, another exophagic species which prefers to bite when most people are outdoors and unprotected [20,24]. *Anopheles darlingi*, a domestic and entirely human-feeding vector, was also rapidly eliminated in Guyana by three years of IRS with DDT, leaving *Anopheles triannulatus*, *Anopheles aquasalis* and *Anopheles albitarsis*[25]. In nearby Suriname, both *An. darlingi* and *Anopheles nuneztovari* appear to have been eliminated by recent scale-up of ITNs [26].

Many mosquito taxa are remarkably robust to intervention scale-up because they exhibit impressive levels of phenotypic plasticity of the synanthropic behaviours that also make them efficient malaria vectors. The best examples of vulnerability to ITNs and IRS relate to vectors that inflexibly express behavioural phenotypes which expose them to insecticide contact, presumably because these traits are deeply “hard-wired” into their genomes through long association with human hosts [27,28]. Historical studies of *An. funestus* in East Africa describe spectacular rigid and absolute preference for humans over animals, even ignoring cattle when they outnumber humans by ten-fold [29]. It is hardly surprising that they were so readily decimated by IRS in this region during the GMEP era [10]. However, most vectors exhibit far greater plasticity of host preference, can obtain blood from animals where they are available [30,31], and are far less vulnerable to control with IRS and ITNs that only protect human blood sources [32]. Covering humans with nets, or any other personal protection measure, reduces the rate of feeding upon people so the proportion of blood meals obtained from humans inevitably drops if any acceptable alternative hosts are present. The resulting drop in the human blood index of blood-fed mosquito samples is greatest among vectors with the greatest preference for animals in settings where those preferred hosts abound, and is exacerbated by physical barriers and repellent pesticides that deter, rather than kill, mosquitoes [33,34]. This phenomenon has been demonstrated dozens of times in the field [33,35], and can occur instantaneously without necessarily requiring any genetic adaptation by the vector.

Across Africa, the timing of biting activity to coincide with human sleeping patterns appears to be a far more important determinant of vector population vulnerability to ITNs than actual preference for feeding indoors or outdoors *per se*[3,5]. Correspondingly, substantial changes in observed biting times of *An. funestus* have been observed following recent scale-up of ITNs in west Africa [36]. The inability of *Anopheles gambiae sensu stricto* to cope with low humidity [37], most probably limits the plasticity with which it can adjust its nocturnal biting activity patterns to avoid ITNs. By comparison, desiccation-tolerant *Anopheles arabiensis*[37] commonly evades contact with IRS and ITNs by feeding in the early evenings when humans are outdoors [9,38,39] and the air remains relatively warm and dry. Within these two species, genetic variability in climatic adaptability may also drive differential vulnerability to IRS [40,41]. Such heritable phenotypic plasticity allows individual mosquitoes to flexibly adapt their behaviour according to the fine-scale environmental conditions they encounter on a day-to-day basis. As a result, the observed behavioural outcomes may well change in response to intervention scale-up without necessarily reflecting any change in the innate preferences of the vector population through genetic selection [42,43]. For example, large proportions of mosquitoes that approach houses, with the intention of entering and feeding upon the occupants, are either killed or deterred by IRS and ITNs. Those that survive obviously persist in their search for blood over more extended periods [44] so that a greater proportion of the remaining vector population may exhibit host-seeking behaviours outside of their normal, preferred peak hours of activity.

## Presentation of the hypothesis

Many of the recently observed reductions in the frequencies of physiological susceptibility [14,45,46] and behavioural vulnerability phenotypes [9,20,47] within residual transmission systems, can therefore be readily explained without assuming any selection for physiological or behavioural resistance traits within the distinct taxa that comprise them. This hypothesis is illustrated numerically through simulations of an African malaria transmission system facing increasing ITN coverage, using an established mathematical model with fixed parameters for the behavioural vulnerability and physiological susceptibility traits of the contributing vector species.

All simulations were executed as previously described [34] with equal baseline emergence rates (E_0_ = 2 × 10^7^ mosquitoes per year) and individual attack availability rates of unprotected humans (a_h,u_ = 1.2 × 10^-3^ attacks per person per host-seeking mosquito per night) for the two vector species and equal numbers of cattle and humans (N_c_ = N_h_ = 1000). All ITN-induced mortality was assumed to occur before feeding so the excess proportion of mosquitoes which are killed after feeding upon a protected human was assumed to be negligible (θ_μ,post_ = 0). The simulated *An. gambiae* and *An. arabiensis* populations differed only in their parameter values for the proportion of human exposure to bites that occurs indoors (π_i_ = 0.9 versus 0.4, respectively [9,38]), the attack availability rates of cattle (a_c_ = 2.5 × 10^-5^ versus 1.9 × 10^-3^ attacks per person per host-seeking mosquito per night [34] and the excess proportions of mosquitoes which are diverted (θ_Δ_ = 0.2 versus 0.6, respectively) or killed before feeding (θ_μ,pre_ = 0.8 versus 0.6 while attempting to attack a human while using an ITN [18,48].

**Figure 1:**
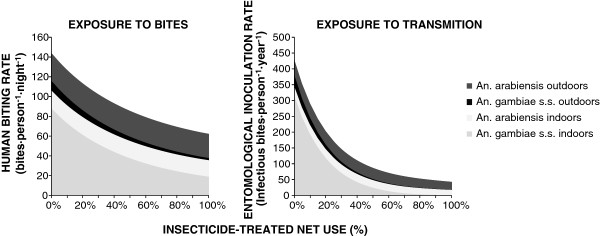
**Simulated decline of indoor and outdoor exposure to bites and to malaria transmission by a mixed population of *Anopheles gambiae s.s. *and *Anopheles arabiensis *as usage rates of insecticide-treated nets (ITNs) increases.**

**Figure 2:**
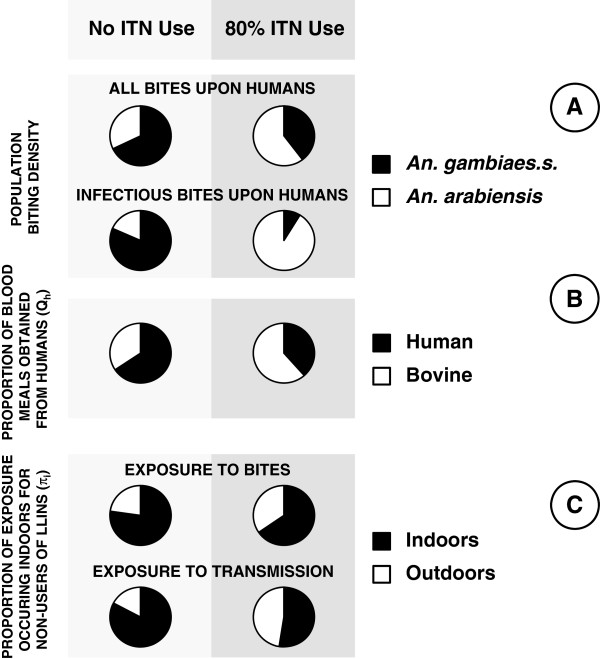
**The impact of high ITN use on the behavioural characteristics and population composition of a transmission systems comprised of a mixed population of *An. gambiae *and *An. arabiensis*.** The relevant summary outcomes presented reflect the means weighted by relative population size or the relative contributions of these two vector species: **A**; Sibling species composition, **B**; Proportion of blood meals taken from humans, and, **C**; Proportion of human exposure to mosquito bites and malaria transmission occurring indoors.

Figure 1 illustrates a simulated baseline scenario with an equal mixture of *An. gambiae* and *An. arabiensis* as an example of a typical historical scenario in the east African settings we are familiar with. *An. gambiae* dominates human exposure to both mosquito bites and malaria transmission before the introduction of ITNs, simply because it feeds almost exclusively upon humans whereas the latter is at least equally likely to feed upon cattle [29,49]. The lower behavioural vulnerability of *An. arabiensis* means it is less likely to make fatal contact with nets and causes its proportional contribution to human biting exposure to grow, from a minority of the human-biting vector population in the absence of ITNs, to the majority following successful scale up (Figures 1 and 2A). This is consistent with recent field observations [11,50] showing that the proportional contribution of *An. arabiensis* to transmission dramatically increases as ITNs are extensively used.

As a result of the increased relative (but reduced absolute) abundance of *An. arabiensis* (Figure 2A), the overall proportion of vector blood meals which is obtained from humans are reduced [51] to approximate the lower values often observed for this species (Figure 2B) [29]. The altered species composition of the residual vector population also influences where and when human exposure to mosquito bites occurs (Figure 2C). Consistent with recent field observations, the impact of ITNs upon the proportion of all bites which occurs indoors is relatively modest [9,38], but it should be noted that the predicted impact upon the proportion of infectious bites occurring indoors is more dramatic, approximating to that of *An. arabiensis*.

These simulations illustrate how reduced frequencies of vulnerable traits among vector mosquitoes in residual transmission systems may, counter-intuitively, reflect intervention success rather than failure (Figures 1 and 2). By definition, the mosquitoes that are most effectively controlled with a given intervention will always be least represented in surveys of the residual populations that persist following scale up.

Consistent with several other contemporary theoretical studies [52,53], all the predicted changes in host-seeking outcomes (Figures 1 and 2) are attributable to the phenotypic plasticity of *An. arabiensis* in particular, and none of these models assume any genetic adaptation of the vector population through heritable alterations of host preference.

## Testing the hypothesis

In order to test this hypothesis, the existing evidence base was reviewed to identify any unambiguous examples of altered frequency of innate behavioural preferences of taxonomically homogenous wild malaria vector populations following IRS or ITN scale-up.

Several reports of apparent change in mosquito behaviour can readily be explained by changes in species composition of the vector population, rather than any heritable modifications of the handful the taxa that contribute to persisting transmission. For example [9], the change in distribution of human exposure to a mixture of members of *An. gambiae* complex reported from Tanzania can probably be attributed to the apparent selective suppression of *An. gambiae* by ITNs, leaving a transmission system dominated by *An. arabiensis.* Similarly, the *An. gambiae* population in Bioko Island [47], was originally composed of two distinct M and S molecular forms that appear to have been differentially affected by IRS [54] and then ITNs so that only the M form remains [47]. Looking further back to the dawn of cytogenetics at the end of the Global Malaria Eradication Programme (GMEP), it was clearly established that the impact of IRS with propoxur upon vector densities varied at village-level geographic scales and was very much dependent upon pre-spray baseline proportions of samples from the *An. gambiae* complex which were caught resting or feeding indoors, as well as their mean biting time [55]. These behaviours were subsequently proven to differ between *An. gambiae* and *An. arabiensis*, making the latter less vulnerable to control with IRS [41]. Recent observations from East Africa indicate that *An. arabiensis* can also adeptly enter and leave houses without exposing themselves to IRS or ITN formulations of pesticides to which they are fully physiologically susceptible [18,48]. This form of behavioural plasticity, avoiding contact with ITNs or IRS wherever they are encountered indoors, pre-dates community-wide scale up of these interventions [18] and so cannot be accurately classified as behavioural resistance in the strict sense [14,56]. It is particularly notable that similar pre-existing traits, specifically short resting times within houses, were identified as the primary obstacle to elimination of malaria transmission by *An. nuneztovari*, *An. darlingi* and *Anopheles punctimacula* in the Americas during the GMEP [16]. The only report of changes in the distribution of biting across the night for a single taxon proven to lack detectable genetic differentiation [42] relates to *An. farauti* in the Solomon Islands [43]. However, it remains to be proven whether this truly reflects alterations in heritable vector behaviours or simply their altered phenotypic expression in an environment with widespread coverage of vector control measures.

A worrying recent study in Benin reported apparently negligible impact upon malaria transmission by universal coverage schemes for pyrethroid-based ITNs, as well as their supplementation with carbamate-based IRS and insecticide-treated wall linings (ITWL) relative to a reference group of villages receiving only targeted coverage with ITNs [57]. Although these vector populations exhibit high levels of physiological resistance to pyrethroids, they are completely susceptible to carbamates and exhibited slightly increased preference for feeding outdoors where ITNs were supplemented with either IRS or ITWL [57]. As detailed quantitative surveys of feeding and resting behaviours by locally important vectors have not yet been reported, the underlying reasons for lack of incremental impact remain unclear [58]. While it is plausible that gaps in biological coverage [32] arose from behavioural avoidance traits such as those discussed in details above [15,17,32], more impressive impacts of supplementary IRS with bendiocarb are apparent elsewhere in Benin [59] and alternative explanations include poor persistence and surface coverage [58]. In the case of the contrast between targeted and universal coverage with ITNs, it must be noted that the improvements in usage achieved by the latter were quite modest [57] and may well offer the most parsimonious explanation for lack of incremental impact upon malaria transmission.

A more worrying recent report from Senegal does raise strong, substantive concerns about the weak impact of vector control, and even rebounding mosquito populations, associated with behavioural and physiological resistance [45]. While it is plausible that epidemiologically relevant behavioural resistance traits have genuinely been selected for in this setting following ITN scale up, significant ambiguity remains because the most relevant vector behaviours have only been partially characterized and all the above interpretational caveats arising from taxonomically selective population suppression and behavioural plasticity may well apply. The extent to which growing frequencies of behavioural and physiological resistance contribute to the observed rebound of transmission remains, therefore, to be determined in this setting.

To conclude, it is clear that intervention-mediated selection for behavioural resistance in the strict sense, meaning an increase in the frequency of heritable behaviour traits in taxonomically homogenous populations which enable them to evade fatal contact with insecticides [14,56], is of great concern but has yet to be conclusively demonstrated in wild vector populations. The contemporary evidence base does not yet provide any clear-cut example of true behavioural resistance and is therefore consistent with the hypothesis presented.

## Implications of the hypothesis

No entomological survey can measure the physiological or behavioural characteristics of dead mosquitoes that have been removed by successful intervention programmes Caution should therefore be exercised before over-interpreting most existing reports of increased frequency of behavioural and physiological resistance traits: this may simply be the result of suppressing the most physiologically susceptible and behaviourally vulnerable of the vector taxa that constituted the original transmission system. Furthermore, none of these field studies can unambiguously attribute these observations to altered frequencies of heritable behavioural preference traits, rather than altered expression of phenotypically plastic behavioural traits in an environment that has been changed by intervention coverage. The importance of plasticity in anthropophagic, endophagic and endophilic behavioural preferences in stabilizing malaria transmission against intervention efforts has long been appreciated [15,17,33,60] and the succinct conclusions of Elliot towards the end of the GMEP appear to be as relevant today as they were four decades ago:

*Delays in malaria eradication programmes are caused more by non-response of fully susceptible vectors to attack measures than by physiological resistance, though the latter receives more attention*[16]*.*

Greater terminological caution is therefore warranted in relation to use of the terms *modification*, *adapt*, *shift* and *resistance* in relation to reports of apparent changes in mosquito behaviours. The term *resilience*, as applied to humans [61-63] and ecosystems [64] may, therefore, be more appropriate for describing pre-existing behaviours that result in evasion of insecticide contact, rather than *resistance* which infers increasingly ability to do so [14,56].

Although the contributions of behavioural and physiological resistance to apparent vector population rebound in Senegal remain unclear, there is no reason to doubt the evidence [45] that this has genuinely occurred. There is clearly no room for complacency but there are also good reasons to be optimistic that well-monitored vector populations can be managed, even to the point of local extinction [10,22,24,25] so long as appropriate tools are available that are well matched to their physiological and behavioural characteristics [14,15,32,65]. For example, the rebound of both *An. funestus* and malaria transmission in South Africa was clearly associated with emergence of physiological resistance to pyrethroids [23], but was effectively tackled by re-introducing DDT [66]. Both examples of vector population and malaria transmission rebound clearly illustrate that such events can only be conclusively documented by longitudinal monitoring of vector population size, the inoculation rates they mediate, and the resulting infection burden among humans. Consistently, continuously and intensively monitored entomological surveillance sites are therefore critical to monitoring, evaluation and planning effective malaria control now and in the future. A particularly important additional reason to monitor and account for behavioural phenotypic plasticity is that it allows organisms to not only cope with population stress in the short-term, but also to evolve more robust adaptive traits in the longer term [67-69]. In the specific case of malaria vectors, recent modelling studies [70] have illustrated how gaps in ITN coverage, including those generated by outdoor feeding behavioural resilience traits [32], can accelerate the equilibration or fixation of physiological resistance alleles. Regardless of whether evasive behaviours observed represent pre-existing resilience or emerging resistance, these will need to be quantified and then targeted with appropriately designed novel interventions that take vector control outside of houses [15,32,65].

Ongoing national or regional entomological monitoring surveys of physiological susceptibility [14,71] should, therefore, be supplemented with biologically and epidemiologically meaningfully [32] estimates of behavioural resilience and resistance phenotypes [9,18,20,29,36,47,49,72], to design, select and optimize the implementation of vector control measures [3-5,15,24,32]. Beyond standardized physiological susceptibility assays of mosquitoes trapped within small artificial containers [14,71], experimental hut surveys [18,73,74] are required to more realistically estimate entry, exit, resting, host attack, and mortality parameters within houses under near-natural conditions [18,73,74]. Furthermore, measurements of human biting rates both indoors and outdoors throughout the night need to be combined with surveys of human behaviour to estimate the proportion of human-vector contact which occurs indoors [4,38]. While alternative methods for quantifying mosquito-human interactions indoors and outdoors are not yet ready to replace human landing catches [75], recent evidence suggests that participants protected with drug prophylaxis are actually safer from malaria than they would be asleep at home [76]. Feeding upon non-human hosts limits malaria transmission [31] but also, creates large gaps in biological coverage of human-targeted interventions like ITNs and IRS [32]. The human blood index remains as important today as it was during the GMEP and can be measured as the proportion of blood meals which are of human origin among samples of resting mosquitoes [30] or inferred using simple models of mosquito host-seeking behaviours parameterized with competitive host choice assays [29,49] and host census data [29]. Recent advances in the application of quality-assured, community-based (CB) trapping schemes greatly improve the scalability, practicality and affordability of continuous survey [77], so it may now be feasible to continually monitor the influence of behavioural and physiological resistance phenotypes upon malaria vectors and transmission on programmatic scales.

While such entomological parameters can be monitored prospectively, they can only be used to infer the suppression or rebound of malaria vectors and transmission where appropriate retrospective baseline data are also available [4,32]. Such legacy data are needed to not only allow the frequency of these phenotypes to be compared, but also the population size of each vector taxon which was historically important [8,9,11,20,25,45]. Settings with little or no coverage with ITNs or IRS are now becoming increasingly rare and misrepresentative so it has never been more urgent to establish sentinel sites for longitudinal, integrated monitoring of vectors populations and the epidemiological events they mediate. While historical literature and data have significant limitations of scope and methodology, they may nevertheless represent the only representative retrospective view of baseline conditions before the recent roll out of ITNs and IRS in many contexts [5].

A suggested generic plan for strengthening national or regional malaria vector monitoring platforms to incorporate assessment of essential behavioural phenotypes and their influence upon vector control impact, mosquito population dynamics and epidemiological outcomes.

1. Expand and consolidate any existing national network of sentinel surveillance sites for physiological resistance of malaria vector mosquitoes to insecticides, ideally integrating with similar platforms for other common mosquito-borne diseases, such as lymphatic filariasis. Such sites should also overlap both with existing historical entomological study sites for which baseline legacy data is available, and with national platforms for assessing malaria burden through cross-sectional malaria indicator surveys or quality-assured facility-based surveillance.

2. Establish an affordable, practical longitudinal community-based (CB) mosquito trapping scheme [77] with a single sampling cluster [78,79] at each of sites for physiological resistance surveillance so that the range of seasonal trends in malaria transmission and contributing vectors (including dry-season minima[56,80]) as well as the impact of national vector control strategies upon these trends can be assessed. Given the diversity of vector species and behaviours across the tropics, this may require initial pilot evaluations to select and calibrate suitable trapping methods or to validate calibrations from elsewhere. Even in Africa, trapping methodologies are poorly standardized [81] and Centers for Disease Control light traps placed beside occupied bednets indoors appear to be the only widely-evaluated exposure-free trapping method with reasonably high relative sensitivity in a diversity of settings [82,83]. However, even this widely accepted method does not function with satisfactory efficacy in some locations [78] and the only trapping tool that has been successfully applied through affordable, quality-assured CB trapping schemes is the relatively new Ifakara Tent Trap [77] which has only been evaluated in two countries [78,79].

3. Given the reliance of scalable CB trapping schemes upon essentially unsupervised field-

4. based personnel, it is also essential establish a quality assurance system in which each of these sites is regularly and randomly re-surveyed by a centrally coordinated, specialist entomological team using the same trapping methods. It is essential that the CB personnel are unaware of the re-survey schedule so that the quality of CB sampling assessed is representative of that implemented all year round.

5. Establish experimental hut capacity at one or two of these sentinel sites, chosen so that most nationally-relevant or regionally-relevant vector species are available at useful densities for as much of the year as possible, enabling the efficacy of vector control interventions to be assessed before and after their introduction [18].

6. Incorporate surveys of vector feeding and resting behaviours, using human landing catch by participants protected with drug chemoprophylaxis [76] and backpack aspirator/screening barrier sampling tools [84,85], respectively, into these quality assurance surveys to quantify the extent to which each important vector species feeds on humans, feeds indoors or rests indoors.

7. Integrate questions relating to relevant human behaviours [5], vector control coverage and livestock ownership into overlapping malaria indicator surveys or, where these do not exist, establish a rolling system of rapid surveys of the human population so that the contributions of vector behaviours, human behaviours and intervention availability to gaps in biological coverage [32] can be quantified.

## Abbreviations

CB: Community-based; GMEP: Global malaria eradication programme; IRS: Indoor residual spraying; ITN: Insecticide-treated nets; ITWL: Insecticide-treated wall lining.

## Competing interests

The authors declare that they have no competing interests.

## Authors’ contributions

NJG and GFK conceived this analysis and review. NJG conducted literature search, drafted the manuscript and edited it based on comments from GFK. PPC and GFK contributed strategies for practical monitoring of mosquito population dynamics on national scales. GFK formulated and implemented the model simulations. All authors agreed to the final version.
